# Offspring sex and parental health and mortality

**DOI:** 10.1038/s41598-017-05161-y

**Published:** 2017-07-13

**Authors:** Øyvind Næss, Laust H. Mortensen, Åse Vikanes, George Davey Smith

**Affiliations:** 1 Epidemiological Division, National Institute of Public Health, Oslo, Norway; 2 Institute of Health and Society, University of Oslo, Oslo, Norway; 3 Department of Public Health, University of Copenhagen, København, Denmark; 4 The Intervention Center, Oslo University Hospital, Oslo, Norway; 5 MRC Integrative Epidemiology Unit (IEU) at the University of Bristol, School of Social and Community Medicine, Bristol, UK

## Abstract

Increased mortality has been observed in mothers and fathers with male offspring but little is known regarding specific diseases. In a register linkage we linked women born 1925–1954 having survived to age 50 (n = 661,031) to offspring and fathers (n = 691,124). Three approaches were used: 1) number of total boy and girl offspring, 2) sex of the first and second offspring and 3) proportion of boys to total number of offspring. A sub-cohort (n = 50,736 mothers, n = 44,794 fathers) from survey data was analysed for risk factors. Mothers had increased risk of total and cardiovascular mortality that was consistent across approaches: cardiovascular mortality of 1.07 (95% CI: 1.03–1.11) per boy (approach 2), 1.04 (1.01–1.07) if the first offspring was a boy, and 1.06 (1.01–1.10) if the first two offspring were boys (approach 3). We found that sex of offspring was not associated with total or cardiovascular mortality in fathers. For other diseases or risk factors no robust associations were seen in mothers or fathers. Increased cardiovascular risk in mothers having male offspring suggests a maternal disease specific mechanism. The lack of consistent associations on measured risk factors could suggest other biological pathways than those studied play a role in generating this additional cardiovascular risk.

## Introduction

To give birth to and raise children may influence parental health. In contemporary populations post-reproductive mortality is related in a U-shaped manner to number of offspring with lowest risk among those with two or three children^1–4^. Sex composition of the offspring may further influence parental health. Boys may exert greater biological, social and psychological costs than girls to give birth to and to raise, and predict more considerable adverse health consequences in mothers compared to fathers^5^. Studies on offspring sex and mortality or longevity have found mixed results. They have covered various historical and epidemiological eras; ranging from settings with a presumably higher proportion of infectious disease deaths in pre-industrial populations, to predominantly non-communicable disease morality in modern populations.

Helle *et al*. found that shorter maternal lifespan was related to the number of sons born^6^. Several studies based on historical populations have produced mixed results^2, 7–12^, whereas studies using more contemporary populations have generally not supported this finding^13–15^. The lack of consistency in results may have several explanations: First, the studies used a wide range of periods with various likely different mechanisms involved^16^. Secondly, previous studies have looked at general outcomes and not outcomes or risk factors reflecting specific potential mechanisms involved. Finally, offspring sex composition is correlated with parity known to be a strong determinant for mortality. And without analytical attention to parity, it is difficult to know if the observed health consequences of offspring sex composition, is predominantly driven by this confounder.

There is some supporting evidence in other studies: In non-human mammals, such as the Red Deer, maternal investment is often greater in male than in female offspring, which may provide an evolutionary advantage as the chance of reproduction for males depends on maternal investment^17^. In humans, sex-differences in several physiological mechanisms suggest that sons impose greater costs on mothers than daughters: boys have a faster rate of fetal growth and are heavier at birth^18^. In monkeys a higher demand for breastfeeding has been observed^19^. Additionally, offspring born subsequent to male siblings have lower birth weight^20^, increased risk of stillbirth^21^, fewer surviving siblings^22^ and lower height in adulthood^23^. Altogether, this suggests that giving birth to boys depletes maternal reserves more than giving birth to girls, and therefore could impact on the mothers’ health.

Here we used several approaches to address the concerns raised above. Associations between offspring sex composition and parental health may arise through direct biological or psychosocial mechanisms. A comparison between mothers and fathers gives indications of gender specific pathways. Associations seen in fathers could come from sharing the same risk environment as mothers, whereas stronger associations seen in mothers might come from the additional contribution of experiencing pregnancy. But it could also come through gender-specific roles in parenting, where offspring sex may influence lifestyle or other concomitants of parenting. One would expect that in the latter case, risk factors or smoking related causes of death, are more prevalent in mothers having more boys. Sex specific cancers, like breast and ovarian cancer are related to parity probably more directly through hormonal and other biological changes in pregnancy, and we would expect greater risk associated with having boys if this mechanism is important^24, 25^. Maternal exposure to male antigens during pregnancy through the feto-placental unit could increase risk of immune-related disorders^26^. And finally, by using alternative approaches in addition to merely counting number of boys and girls, sex of first offspring and proportion of offspring boys, we would be able disentangle the impact parity may have on the observed associations from offspring sex composition.

We investigated whether offspring sex is related to parental risk factors, cancer risk and cause-specific mortality using a large population sample.

## Methods

### Population

The data were organized into trios. We included only those groups where we had information on the full reproductive age of mothers from 15 to 50 years and with follow-up of death afterwards. Fathers were also required to survive to age 50. Identity of offspring and fathers was available from Norwegian multigenerational data^27^. This gave 1,936,257 offspring with 784,325 mothers and 786,262 fathers. We analysed those with complete data on covariates giving 661,013 women and 691,124 men. 15% women and 12% men were excluded because they lacked information on covariates. Associations were largely similar when analysing the full sample and with the exclusions. The parents were linked to their offspring and to the Cause of Death Registry, the Cancer Registry, Statistics Norway and from the Cohort of Norway (CONOR)^28^. The CONOR participants comprised a sub cohort of the 661,013 mothers and 691,124 fathers with 50,736 women and 44,794 men who participated in one of ten regional, population-based health surveys in the period 1994–2003.

### Exposure, covariates and outcomes

Number of boys, girls and total offspring was calculated for each individual. Length of education was retrieved for each individual from the national educational database as highest achieved by age 30 and categorized into five categories. Length of education was categorized into the following five ordered groups: 7–9 years (representing completion of primary school education only), 10–11 years (middle school), 12 years (secondary school), 12–16 years (college) and >16 years (usually indicating completion of a university degree).

Outcome variables were death by cause (Cause of death registry) and by incident cases of cancer (Cancer registry). Causes of death (ICD-10 codes) were: all causes, cardiovascular causes (I00-I99) and pulmonary cancer and COPD combined (C32-C34 and J40-J47). The following codes were used for cancer coming both from the Cancer Registry and the Cause of Death Registry: breast cancer (C50), ovarian cancer (C56), prostate cancer (C61) and testicular cancer (C62). The cancer analysis was done separately so that cancer events were incident cases and did not include cancer deaths. Autoimmune diseases were retrieved from the Disability Register: rheumatoid arthritis (ICD-10: M05), coeliac disease (ICD-10: K90), Crohn’s disease (ICD-10 K50) and ulcerative colitis (ICD-10: K51).

CONOR assessments on risk factors included information on and measurement of height and weight, fasting lipids, arterial pulse, systolic blood pressure, diabetes, mental distress, current smoking, alcohol consumption frequency past year and physical activity. Diabetes was recorded by the following question: “Do you have or have you had diabetes?” Physical activity assessment included two questions regarding the number of hours per week in the past year spent in light and vigorous (resulting in shortness of breath and or sweating) physical activity. Past-year alcohol consumption frequency was categorized into three groups (≥1 week, 1–3 times a month, and less than monthly which included abstainers). Lipids were measured by an enzymatic method (Roche Diagnostic, Swizterland). The average of the last two of three systolic blood pressure readings taken after a 2 min rest by an automatic device (DINAMAP, Criticon, Tampa, FL, USA) was used in analyses.

### Analytical approach and statistical methods

Disentangling the effect of offspring sex from the effect of parity is difficult. When using number of boys or girls as exposures, this will pick up some of the effect that goes through parity. For this reason we used three strategies and investigated whether these gave coherent findings: Firstly, we estimated the relation between *number of boys* and *girls* and parental mortality. Secondly, we estimated the relation between parental mortality and *sex of first offspring* and the *sex* of *first and second offspring*. For the first two offspring the combination was coded as: girl-girl (reference), girl-boy or boy-girl into a “mixed” category and boy-boy and included those with at least two children (n = 562,677 women and n = 584,515 men). Thirdly, we analysed the *proportion of boys* (number of boys divided by total number of offspring). For the presentation of the results, estimates from the second and third strategy are presented in the main tables and from the first in supplementary tables.

Mortality and morbidity data were analyzed using Cox proportional hazards regression. The proportional hazards assumption was assessed by visual inspection of plots and by testing Schoenfeld residuals. No indication of a violation of the proportional hazard assumption was found. Time was entered as age. The models were adjusted for year of birth. Difference in the parameter estimates between the variables *number of boys* and *number of girls* was tested using the command test in STATA 12 after the Cox regression model was run with both parameters included in the model. Difference in estimates between mothers and fathers for *number of boys* and *number of girls* investigated separately, was tested by pooling mothers and fathers together and fitting interaction terms for sex of the parent and *number of boys* and *number of girls* respectively. For the analysis of risk factors in the sub-cohort of CONOR, linear and logistic regression models were used.

We conducted several additional analysis by linking to data from Social Security Registry and the Medical Birth Registry (births after 1967), see Supplementary Tables 9 and 10. We also investigated CVD and all-cause mortality in mothers having offspring with twins, perinatal deaths, pre-eclampsia and with different length of inter-pregnancy intervals, and we investigated CVD mortality in the CONOR sub-cohort.

### Ethics

The study is part of the study “Inter- and intra-generational transmission of risk” was approved by the Norwegian Regional Ethics Committee, No. 2010/260.

## Results

The sub-cohort was slightly younger than the main cohort (Table 1). *Number of boys* and *number of girls* were each related to earlier year of birth in offspring and parents higher proportion with primary education. High proportion of boys was related to these covariates to a much lesser degree with slightly lower proportion with primary education only. *Sex of first offspring* and *sex of first two offspring* did not vary substantially by these covariates (Supplementary Table 1).

**Table 1:** Some characteristics according to number of boys and girls in the study population.

	N	Year of birth (mean and standard deviation)	Deaths (n)	Year of birth offspring (mean and standard deviation)	Primary education only (%)	Total N° offspring (mean)
Mothers						
Main sample						
At least one offspring	661,013	1941 (9.0)	88,620	1968 (9.2)	67	2.5
At least two offspring	562,693	1941 (8.9)	71,869	1968 (9.0)	67	2.8
Sub-cohort						
At least one offspring	50,736	1945 (10.3)	2,926	1972 (10.8)	60	2.6
At least two offspring	43,860	1945 (10.2)	2,476	1972 (10.6)	60	2.8
Fathers						
Main sample						
At least one offspring	691,124	1939 (10.5)	186,416	1969 (9.6)	55	2.5
At least two offspring	584,515	1939 (10.3)	153,915	1969 (9.3)	55	2.8
Sub-cohort						
At least one offspring	44,794	1941 (11.1)	5,873	1971 (10.6)	51	2.5
At least two offspring	37,816	1941 (11.0)	4,977	1971 (10.4)	51	2.8

**Figure 1:**
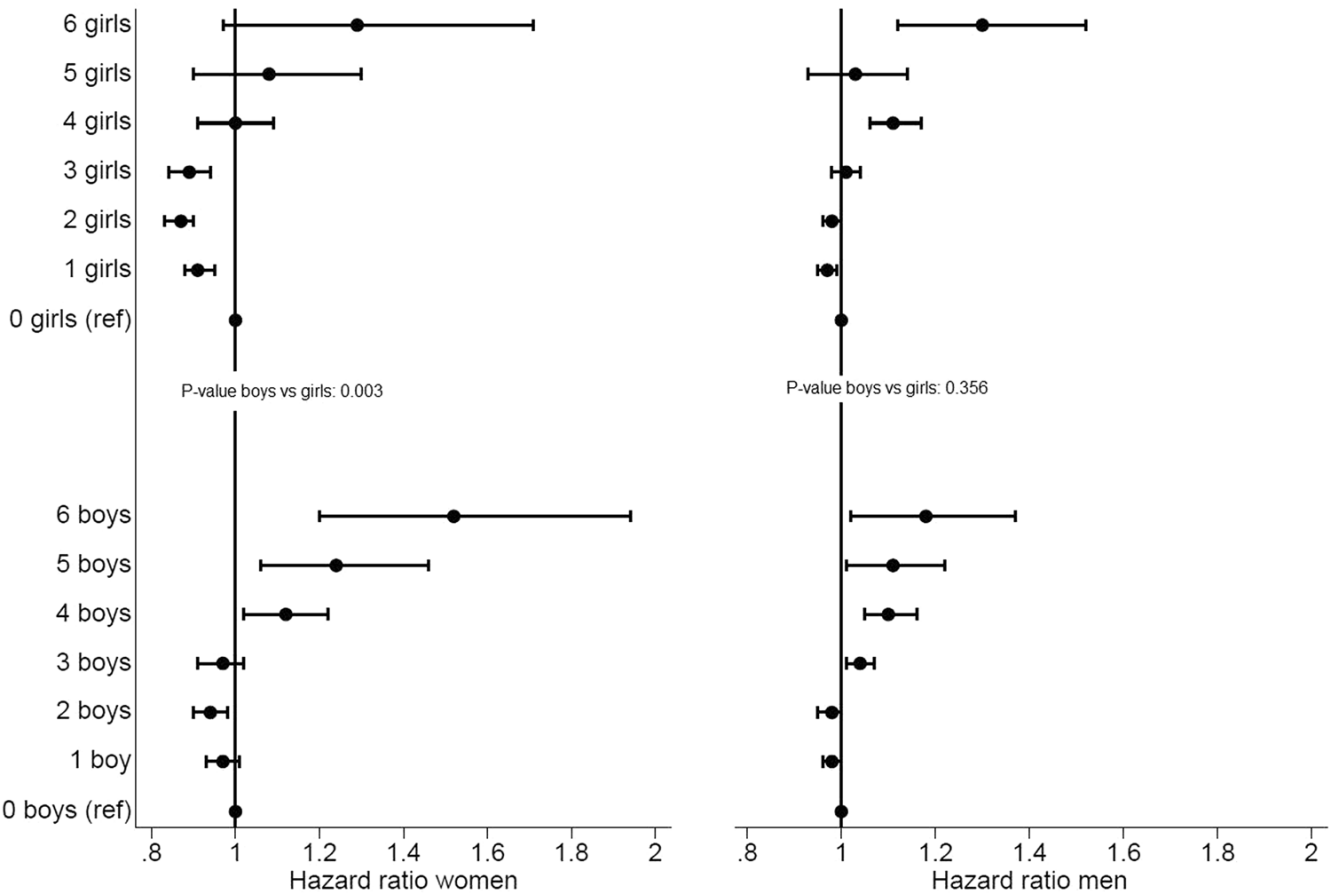
Age adjusted hazard ratio of cardiovascular mortality by number of offspring boys and girls among women (n = 661,013) and men (n = 691,124).

In mothers, there was a U-shaped relation (quadratic term p-value < 0.001) between *number of boys* and *girls* and all-cause mortality with lowest risk with two and three boys or girls (Supplementary Table 2). Cardiovascular causes also showed a U-shaped pattern by *number of boys* in mothers, with increased risk for more than three boys (Fig. 1). Such a pattern was also seen for *number of girls*, though to a lesser degree, and there was a smaller relationship with many girls. For lung cancer and COPD there was a small inverse relationship for both *number of boys* or *girls*.

In fathers the U-shaped pattern was less pronounced, there was little increased risk of all cause death and an increased risk in cardiovascular death by both *number of boys* and *girls* which was largely attenuated when including number of other sex offspring and length of education (Supplementary Table 3). For lung cancer and COPD there was a small positive relation with *number of boys* and *girls*.

*Sex of the first offspring* was associated with higher risk of all cause and cardiovascular cause mortality in women (Table 2). The *sex composition of first two* offspring was associated with hazard ratio of 1.06 (95% CI: 1.01–1.10) higher risk of cardiovascular mortality among mothers when having two boys compared to having two girls. No comparable associations were seen in fathers or for other outcomes when using this approach.

**Table 2:** Age adjusted hazard ratio of cause specific mortality from the Cause of Death Registry (all cause, circulatory and lung cancer) and incident cancer from the Norwegian Cancer Registry (breast cancer and ovarian cancer) if the first and second offspring sex was boy among fathers and mothers born 1925–54 with follow up of from age 50.

	Hazard ratio (95% CI)						
	At least one offspring^a^ Sex of first offspring			At least two offspring^b^ Sex of first and second offspring			
	Girl (ref)	boy	*p-value* (*trend*)	girl-girl (ref)	boy-girl/girl-boy	boy-boy	*p-value* (*trend*)
*Mothers*							
All causes (n = 93,518)	1.00	1.02 (1.01–1.03)	*0.007*	1.00	1.01 (0.99–1.03)	1.02 (1.00–1.05)	*0.066*
Cardiovascular (n = 22,072)	1.00	1.04 (1.01–1.07)	*0.008*	1.00	1.00 (0.96–1.04)	1.06 (1.01–1.10)	*0.011*
Lung cancer and COPD (n = 11,503)	1.00	1.01 (0.97–1.04)	*0.780*	1.00	0.96 (0.91–1.01)	0.99 (0.94–1.05)	*0.907*
Breast cancer (n = 30,649)	1.00	1.00 (0.98–1.03)	*0.991*	1.00	1.00 (0.96–1.03)	0.99 (0.95–1.03)	*0.626*
Ovarian cancer (n = 6935)	1.00	1.00 (0.95–1.06)	*0.925*	1.00	1.04 (0.96–1.13)	1.02 (0.93–1.11)	*0.783*
*Fathers*							
All causes (n = 196,867)	1.00	1.00 (0.99–1.01)	*0.917*	1.00	0.99 (0.97–1.00)	1.00 (0.99–1.02)	*0.819*
Cardiovascular (n = 75,557)	1.00	1.00 (0.99–1.02)	*0.624*	1.00	0.99 (0.97–1.01)	1.00 (0.98–1.03)	*0.738*
Lung cancer and COPD (n = 21,994)	1.00	1.00 (0.97–1.02)	*0.763*	1.00	1.01 (0.98–1.05)	1.01 (0.96–1.05)	*0.726*
Testicular cancer (n = 1948)	1.00	0.97 (0.89–1.06)	*0.487*	1.00	1.06 (0.93–1.20)	1.08 (0.94–1.24)	*0.285*
Prostate cancer (n = 31,827)	1.00	1.00 (0.97–1.02)	*0.920*	1.00	1.00 (0.97–1.03)	0.98 (0.95–1.02)	*0.340*

^a^n = 661,013 for women and n = 691,124 for men. Adjusted for year of birth, education and parity.

^b^n = 562,677 for women and n = 584,515 for men. Adjusted for year of birth, education and parity.

Both *number of boys* and *girls* were inversely related to risk of breast and ovarian cancer in mothers (Supplementary Table 6). Adjusting for covariates had minimal impact on the estimates, and the estimates were similar for *number of boys* and *number of girls*. The *proportion of boys* was related to all-cause mortality and cardiovascular mortality in mothers and not in fathers (Table 3).

**Table 3:** Age adjusted hazard ratio of death among mothers and fathers by proportion of boys among all and stratified by number of offspring.

	Hazard ratio (95% CI)					
	Mothers (n = 661,013)			Fathers (n = 691,124)		
	All cause	CVD	Lung cancer and COPD	All cause	CVD	Lung cancer and COPD
Total	1.03 (1.01–1.05)	1.07 (1.03–1.12)	1.02 (0.97–1.08)	1.00 (0.99–1.01)	1.01 (0.99–1.03)	0.99 (0.95–1.03)
Total *adjusted* ^a^	1.03 (1.01–1.05)	1.07 (1.03–1.11)	1.02 (0.97–1.08)	1.00 (0.99–1.02)	1.01 (0.99–1.03)	0.99 (0.96–1.04)
By N° offspring:						
1	1.03 (1.00–1.07)	1.06 (1.00–1.14)	1.07 (0.97–1.17)	0.99 (0.97–1.02)	1.02 (0.99–1.06)	0.97 (0.91–1.04)
2	1.01 (0.97–1.04)	1.06 (0.99–1.13)	0.98 (0.89–1.07)	0.99 (0.97–1.02)	0.98 (0.94–1.02)	1.02 (0.96–1.10)
3	1.04 (0.99–1.10)	1.10 (1.00–1.21)	1.02 (0.90–1.16)	1.02 (0.99–1.06)	1.05 (1.00–1.11)	0.99 (0.90–1.08)
4	1.04 (0.96–1.12)	1.01 (0.87–1.18)	0.92 (0.74–1.14)	1.01 (0.96–1.07)	1.03 (0.95–1.11)	0.97 (0.84–1.12)
5	1.10 (0.95–1.27)	1.20 (0.92–1.56)	1.35 (0.91–2.00)	1.10 (1.00–1.20)	0.96 (0.83–1.11)	1.24 (0.96–1.60)
6 or more	1.21 (0.98–1.50)	1.11 (0.77–1.60)	1.31 (0.71–2.44)	0.92 (0.80–1.05)	1.01 (0.82–1.24)	0.70 (0.47–1.03)
*P-value* (*interaction*)	*0.375*	*0.885*	*0.730*	*0.196*	*0.910*	*0.854*
By year of birth						
1925–1929	1.04 (1.01–1.07)	1.08 (1.02–1.14)	1.10 (1.00–1.20)	1.00 (0.99–1.02)	1.01 (0.96–1.05)	0.96 (0.89–1.04)
1930–1939	1.04 (1.00–1.07)	1.06 (0.99–1.14)	0.95 (0.87–1.04)	1.02 (0.99–1.05)	1.02 (0.97–1.06)	0.99 (0.99–1.07)
1940–1949	0.99 (0.94–1.04)	1.08 (0.95–1.24)	1.01 (0.89–1.14)	0.98 (0.94–1.02)	0.99 (0.92–1.07)	1.02 (0.91–1.14)
1950–1958	1.03 (0.92–1.15)	1.01 (0.72–1.40)	1.21 (0.91–1.61)	0.95 (0.86–1.05)	0.95 (0.78–1.16)	0.94 (0.70–1.27)
*P-value* (*interaction*)	*0.255*	*0.469*	*0.997*	*0.002*	*0.166*	*0.025*

^a^Fixed effect analysis adjusting for the effect of belonging to any of the parity group strata.

We found no association with offspring sex in the following autoimmune diseases: rheumatoid arthritis, coeliac disease, Crohn’s disease and ulcerative colitis (Table 4). In mothers in the sub-cohort of CONOR most risk factors were related to unfavorable levels for both number of boys and number of girls (Supplementary Tables 4 and 5). Percent daily smokers and percent drinking more than 2 units of alcohol per week decreased with *number of boys* and *girls*. In fathers the same pattern was seen, except for triglycerides and cholesterol (boys), and mental distress and daily smoking (boys and girls). Neither *sex of first offspring* nor *sex of first two offspring* was related to any of these risk factors in both fathers and mothers (Tables 5 and 6). ‘*Proportion boys’* was also not related to any of these except for physical inactivity in fathers.

**Table 4:** Age adjusted odds ratio by some common autoimmune diseases among mothers and fathers by offspring birth composition.

	Odds ratio (95% CI)							
	Mothers (n = 661,013)^a^				Fathers (n = 691,124)^a^			
	Reumatoid arthritis	Coliac disease	Ulcerous colitis	Mb Crohn	Reumatoid arthritis	Coliac disease	Ulcerous colitis	Mb Crohn
Per boy	0.91 (0.82–1.00)	0.96 (0.81–1.13)	1.15 (0.85–1.55)	0.98 (0.60–1.61)	0.95 (0.78–1.16)	0.84 (0.67–1.06)	0.76 (0.57–1.01)	1.16 (0.59–2.27)
*Mothers vs fathers* ^a^	*0.674*	*0.379*	*0.049*	*0.693*	—	—	—	—
First two offspring								
Girl-girl	1.00 (ref)	1.00 (ref)	1.00 (ref)	1.00 (ref)	1.00 (ref)	1.00 (ref)	1.00 (ref)	1.00 (ref)
Girl-boy or boy-girl	0.98 (0.89–1.08)	1.01 (0.86–1.19)	0.87 (0.64–1.18)	0.98 (0.60–1.61)	0.98 (0.82–1.19)	0.99 (0.80–1.22)	0.99 (0.75–1.30)	0.75 (0.40–1.43)
Boy-boy	0.95 (0.85–1.06)	0.97 (0.81–1.16)	1.20 (0.87–1.66)	0.97 (0.55–1.72)	0.96 (0.77–1.19)	0.84 (0.66–1.08)	0.92 (0.67–1.27)	0.84 (0.40–1.73)
*Trend* (*p-value*)	0.379	0.710	0.207	0.928	0.694	0.164	0.608	0.633
*Mothers vs fathers* ^a^	*0.962*	*0.371*	*0.204*	*0.742*	—	—	—	—

*p-value* ^a^.

**Table 5:** Mean values (sd) or percentages with test of differences (linear or logistic regression) of some risk factors among mothers in a linked sub cohort being part of the Cohort of Norway (CONOR) according to sex of first offspring, sex of first two offspring and proportion boys.

	Age at examination (sd)	BMI (kg/m ^2^)	Chol- sterol (mmol/l)	Systolic Blood pressure (mmHg)	Diabetes (%)	Mental distress (%)	Daily smoker (%)	Alcohol (<2units per week)	Physical Inactivity (%)	Family history of CHD (%)	Previous CVD (%)	N
Mothers												
*Sex of first offspring* ^a^												
Girl	52 (10.6)	26.0 (4.5)	5.99 (1.2)	133 (21.1)	2.5	5.3	34.1	8.8	6.2	50.1	4.5	24,658
Boy	52 (10.5)	26.0 (4.5)	5.98 (1.2)	133 (21.0)	2.4	5.5	34.1	8.9	6.5	50.1	4.7	26,078
P-value	0.828	0.821	0.392	0.962	0.358	0.487	0.897	0.320	0.143	0.978	0.198	
*Sex of first two offspring* ^b^												
Girl-girl	52 (10.4)	26.0 (4.5)	5.97 (1.2)	133 (21.0)	2.5	5.1	33.5	8.2	6.3	50.0	4.5	10,376
Girl-boy or boy-girl	52 (10.5)	26.0 (4.4)	5.99 (1.2)	133 (21.0)	2.4	5.3	33.3	8.8	6.0	50.0	4.5	21,953
Boy-boy	53 (10.5)	26.0 (4.4)	6.00 (1.2)	133 (21.0)	2.4	5.1	33.4	8.5	6.4	49.7	4.8	11,531
P-value	0.387	0.939	0.121	0.722	0.497	0.863	0.924	0.565	0.828	0.503	0.319	
*Proportion boys*												
<0.5	52 (10.6)	26.0 (4.5)	6.00 (1.2)	132.9 (21.1)	2.5	5.4	34.1	8.4	6.6	50.4	4.9	19,527
>=0.5	52 (10.5)	26.0 (4.4)	6.00 (1.2)	132.5 (21.1)	2.4	5.4	34.2	9.2	6.2	50.0	4.4	31,209
P-value	0.082	0.645	0.194	0.614	0.846	0.931	0.503	0.781	0.786	0.829	0.257	—
*Missing*	*0*	*137*	*61*	*16*	*631*	*8,469*	*253*	*1839*	*2829*	*2991*	*537*	—

^a^These 50,736 women from the full cohort were identified as participating in CONOR with at least one offspring.

^b^These 43,860 women from the full cohort were identified as participating in CONOR with at least two offspring.

**Table 6:** Mean values and standard deviations with test of differences (linear or logistic regression) of some risk factors among fathers in a linked sub cohort being part of the Cohort of Norway (CONOR) according to sex of first offspring, sex of first two offspring and proportion boys.

	Age at examination (sd)	BMI (kg/m ^2^)	Chol- sterol (mmol/l)	Systolic Blood pressure (mmHg)	Diabetes (%)	Mental distress (%)	Daily smoker (%)	Alcohol (<2units per week)	Physical Inactivity (%)	Family history of CHD (%)	Previous CVD (%)	N
Fathers												
*Sex of first offspring* ^a^												
Girl	56 (11.4)	26.6 (3.4)	6.00 (1.1)	139.2 (19.3)	3.6	3.5	30.1	19.4	6.7	46.2	12.5	21,828
Boy	56 (11.5)	26.6 (3.4)	6.00 (1.1)	139.5 (19.0)	3.7	3.6	30.1	19.7	6.4	46.2	12.5	22,966
P-value	0.689	0.682	0.725	0.196	0.620	0.372	0.849	0.232	0.221	0.980	0.986	
*Sex of first two offspring* ^b^												
Girl-girl	56 (11.2)	26.7 (3.4)	6.02 (1.1)	139.4 (19.5)	3.6	3.3	29.8	19.3	6.8	46.6	12.6	8,900
Girl-boy or boy-girl	56 (11.3)	26.6 (3.4)	6.00 (1.1)	139.3 (19.1)	3.8	3.3	30.6	19.6	6.5	46.2	12.9	19,109
Boy-boy	55.9 (11.4)	26.6 (3.4)	6.00 (1.1)	139.6 (19.1)	3.6	3.6	29.9	19.4	6.2	46.5	12.6	9,807
P-value	0.612	0.497	0.317	0.359	0.620	0.263	0.976	0.849	0.099	0.871	0.951	
*Proportion boys*												
<0.5	56 (11–5)	26.6 (3.4)	6.00 (1.1)	139.0 (19.3)	3.8	3.7	30.8	19.0	6.4	45.9	12.7	17,130
>=0.5	56 (11.4)	26.0 (3.4)	6.00 (1.1)	139.2 (19.1)	3.6	3.5	30.9	19.9	6.6	46.3	12.4	27,664
*P-value*	*0.366*	*0.456*	*0.320*	*0.523*	*0.241*	*0.762*	*0.871*	*0.934*	*0.040*	*0.954*	*0.213*	
*Missing*		*90*	*47*	*32*	*443*	*11,022*	*124*	*1237*	*1621*	*3380*	*284*	

^a^These 44,794 fathers from the full cohort were identified as participating in CONOR with at least one offspring.

^b^These 38,816 fathers from the full cohort were identified as participating in CONOR with at least two offspring.

For the additional analysis we found no increased risk for CVD and all-cause mortality in mothers having offspring experiencing perinatal death, experiencing pre-eclampsia or having different inter-pregnancy intervals. Women with twin births had increased CVD risk (proportion boys gave of 1.51 (1.13–2.03) and sex of first offspring 1.20 (0.99–1.47)) (Supplementary Table 6). In the CONOR sub-cohort CVD hazard ratios were for women 1.07 0.92–1.26) adjusting for year of birth and parity, and 1.07 (0.92–1.26) after adjusting also for smoking, cholesterol, triglycerides, physical inactivity and systolic blood pressure.

## Discussion

Mothers with more male offspring had increased cardiovascular mortality. This was not seen in fathers. For other disease outcomes there was no differential association of offspring sex in mothers and fathers. Interesting patterns of association with risk factors was seen for mothers and fathers, in which both *number of boys* and *girls* were related to unfavorable levels but not with *sex of first* and *first and second offspring*.

### Strengths and limitations

The comprehensive data linkage, size, follow-up, details on causes of death and risk factors in the data are clear advantages. Mortality follow-up took place over many years, and we did not have information on emigration. But we do not consider this to cause bias as only 3–5 per 1000 inhabits emigrated from Norway in this period. The study benefits from triangulating analytical approaches to produce more robust conclusions. It is unlikely that parents having a first offspring boy differ systematically from those who have a first offspring girl. The strength of this triangulation is further supported by the distribution of covariates and risk factors, in which the clear pattern when using number of boys disappears when using *sex of first* and *sex of first and second offspring*. But we would caution against interpreting that there is a further increase of CVD risk in women having more than two boys.

In Nordic registry data it has previously been shown that sex of the first two pregnancies is related to odds for having a third offspring^29^. If the first two offspring are boys, the odds for having a third pregnancy is larger than if the first two pregnancies were girls or mixed. However, this trend started around 1990, and is not likely to influence this cohort. Since parity is a confounder, and if having two boys increases odds of having a third offspring we would expect parity to decrease rather than increase the association with having boys since having mostly girls would be related to increased parity and increased mortality. Sensitivity analyses restricting to those with exactly one and two offspring showed similar estimates. We also looked at risk of having first offspring boy in different birth cohorts: The risks for CVD of having first offspring boy in the different birth cohorts were largely similar: 1.03 (0.99–1.07) 1925–1929, 1.06 (1.01–1.11) 1930–1939, 1.04 (0.95–1.14) 1940–1949 and 0.86 (0.69–1.08) 1950–1955.

The covariates and risk factors appeared more strongly related to *number of boys* or *girls* than to *proportion of boys* or *sex of first offspring* and *sex of first two offspring*. This probably reflects that the exposure variables *number of boys* and *girls* are more directly confounded by family size. Composition of sex in the first and sex of first and second offspring were unrelated to almost any of the covariates or risk factors. In addition to indicating that sex of the first offspring(s) seems to be random, at least when number of offspring is low, it suggests that having boys does not impact much on risk factor profile in parents.

In some countries sex-selective abortion is known to affect offspring sex composition. In Norway voluntary pregnancy termination in most cases takes place before 12 weeks of pregnancy, a time point when sex of the fetus is not known to the parents^30^. Proportion of stillbirths in Norway is comparatively low, 5.9 per 1000 deliveries^31^. Women who experienced offspring death perinatally did not have differential risk. Paternal misclassification could have attenuated the estimate in fathers compared to mothers, but is not likely to differ according to sex of offspring.

### Determinants of sex composition

Biological mechanisms underlying offspring sex is not settled^32^. High caloric intake is known to skew offspring sex ratio towards males in mice, cows and horses^33–35^. Observational studies among humans have shown some variations in sex ratio with conditions of war or poor nutrition but this has not been seen in data from the Dutch famine study rendering little support for a claim that environmental changes will have large impact on the sex ratio^36, 37^. There might be a “selective loss” of males due to susceptibility to environmental insults or nutritional deficiencies consistent with the Trivers-Willard hypothesis that good conditions during pregnancy will promote investment in male fetuses^38^. This mechanism would go in the opposite direction of higher mortality for mothers who have more boys since women in harsh environmental conditions potentially lose male foetuses and these conditions would also be expected to increase their mortality.

High sex specific parental levels of oestrogen and testosterone are associated with having male offspring, whereas high levels of progesterone and gonadotropins are associated with having female offspring^24^. High levels of testosterone are known to have immunosuppressive effects, which may be an advantage since the maternal immune system then is exposed to male-specific antigens^25^. Observations from studies of humans are, however, not unequivocally in concordance with the testosterone theory. Whether the high sex specific parental levels of oestrogen and testosterone at the time of conception remain after birth, and subsequently may trigger maternal risk of CVD, is not known.

### Family factors and causal inference on fertility and health

Given the physiological changes during pregnancy, birth and lactation, women carry the highest direct biological burden in regard to reproduction^1^. Effects seen in fathers may be due to shared family factors both related to number of offspring and later health^39^. The U-shape pattern seen when number of boys and girls were analyzed probably reflects the influence of parity. Father’s health is used as a comparator because a stronger effect in mothers would suggest that factors during pregnancy may be of importance. A review from 2007 concluded that in natural fertility conditions, i.e. when fertility is near its biological maximum, longevity does not decrease when the number of children increases but, in modern populations, mortality could increase when women have more than 5 children^3^. In a Norwegian study covering a similar period, odds of death relative to those for subjects with two children were highest for the childless in both women and men and next highest for those with only one child^40^. The similarity of results for women and men suggests social pathways underlying these associations between reproductive history and health. They also found for 11 causes of death, fertility was related to increased risk, supporting a general explanation for the relationship^5^.

### Explanations for offspring sex and parental health

Our study suggested increased risk of CVD in mothers with boys but not with increased levels of CVD related risk factors. This could suggest risk factors are not involved which is supported by findings in the CONOR sub-cohort where the (imprecisely estimated) greater association in women for CVD was not attenuated after adjusting for important risk factors. In general differential associations of offspring sex with parental health may mechanisms that are biologically or socially informative, even if effect sizes are small.﻿ Sons and daughters may provide emotional, interpersonal, financial and material support to the parents, which may be gendered^41^. Studies in societies with strong preference towards sons, such as China and Bangladesh, are at odds with this, suggesting a context specific association: In Bangladesh there was an increased survival according to number of sons, possibly due to improved parental socioeconomic circumstances consequent on this.

Maternal-fetal exchange of genetic material (fetal microchimerism) has been suggested to have health implications for both the fetus and the mother^42^. Half of the fetal tissue antigens have paternal origin and maternal immune reactions must be suppressed or tolerated to continue the pregnancy^43^. With a male fetus, the maternal immune system is exposed to male antigens that may give rise to long-lasting immunity against male-specific antigens. Such male-specific antigens have been found to give higher risk of graft-versus-host disease in stem-cell transplantation with female donors to male recipients^44^. Such changes affect immune tolerance, energy metabolism and cardio-respiratory function, all triggered by the feto-placental unit^26^. Our study found no reflection of this in rheumatoid arthritis, coeliac disease, crohn’s disease or ulcerative colitis, however.

Micro-vascular vasodilatation is enhanced in women with a male fetus^45^. In preeclampsia, this vasodilatation is reduced in women with a male but not a female fetus, compared to a normal pregnancy. There is also growing evidence that the physiological adjustments do not return to the pre-pregnancy state when pregnancies are complicated by certain diseases, such as preeclampsia and gestational diabetes^46^. These conditions may occur due to pre-existing subclinical disease, later manifest in CVD, autoimmune disease or early maternal mortality, creating an additional burden of disease for the mother. Our sub-analysis on perinatal death, pre-eclampsia, perinatal deaths did not support this.

## Conclusion

Mothers, not fathers, have increased risk of cardiovascular disease when having boys compared to girls. The difference is not reflected in the pattern of conventional cardiovascular risk factors, suggesting that other biological or social pathways may play a role.

## Electronic supplementary material

Figure 1

Supplemental tables
