# Supplementary figures and images for: Offspring sex and parental health and mortality
Source: Sci Rep. 2017 Jul 13;7:5285. doi: 10.1038/s41598-017-05161-y (PMC5509737; doi:10.1038/s41598-017-05161-y)

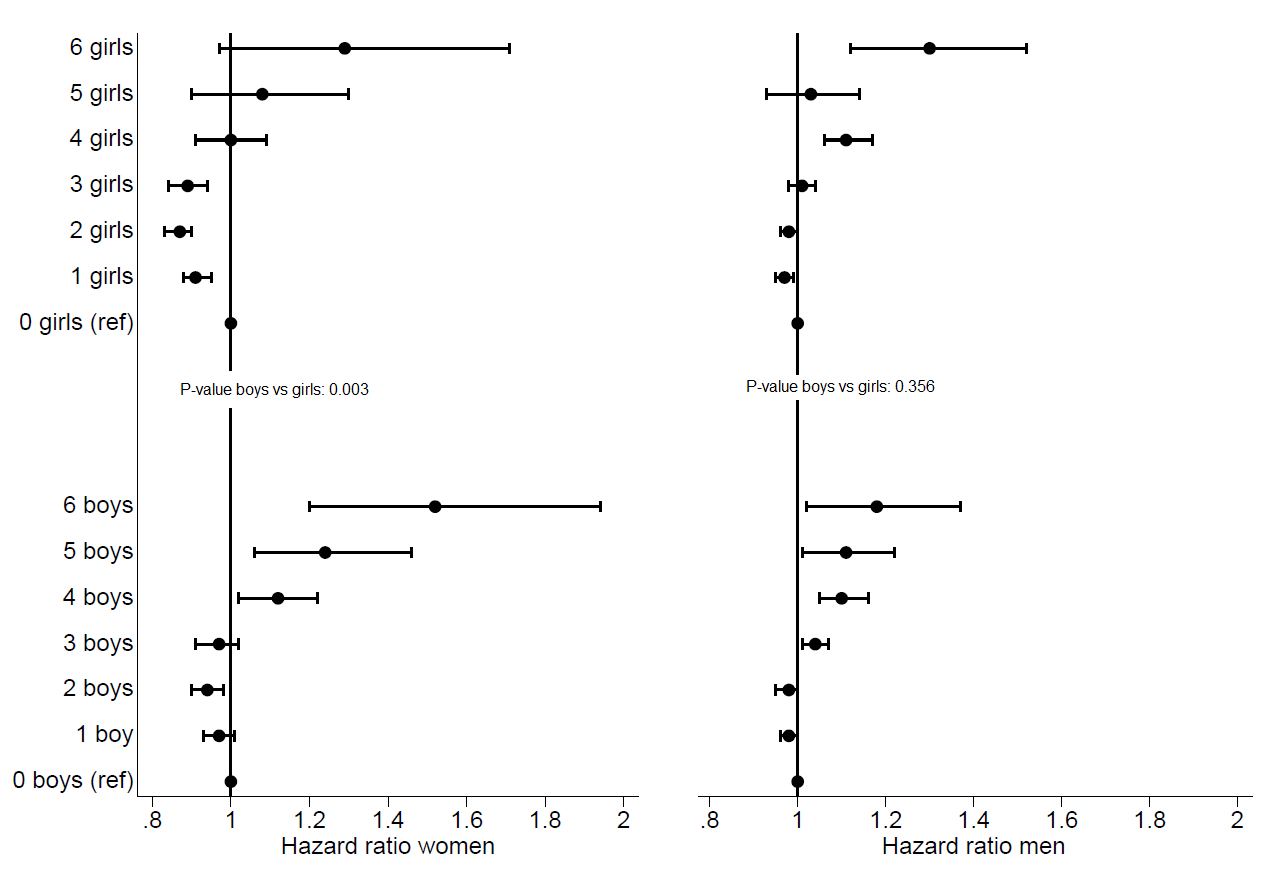

Supplement: Supplementary file 1 — Figure 1 [file 41598_2017_5161_MOESM1_ESM.doc]
