# Supplementary material for: Offspring sex and parental health and mortality
Source: Sci Rep. 2017 Jul 13;7:5285. doi: 10.1038/s41598-017-05161-y (PMC5509737; doi:10.1038/s41598-017-05161-y)
Supplement: Supplementary file 2 — Supplemental tables [file 41598_2017_5161_MOESM2_ESM.doc]

**Offspring sex and parental health and mortality**

A family based study of post reproductive risk factors, cancer incidence and cause specific mortality

Øyvind Næss (1, 2), Laust H Mortensen (3), Åse Vikanes (1, 4), George Davey Smith (5)

1. Epidemiological Division, National Institute of Public Health, Norway
2. Institute of Health and Society, University of Oslo, Norway
3. Department of Public Health, University of Copenhagen, Denmark

(4) The Intervention Center, Oslo University Hospital, Oslo, Norway

(5) MRC Integrative Epidemiology Unit (IEU) at the University of Bristol, School of Social and Community Medicine, UK

SUPPLEMENTARY TABLE 1. Some characteristics of the population according to proportion boys and sex of first two offspring

|  | N | Year of birth (mean) | Deaths  (n) | Year  of birth offspring  (mean) | Primary  Education  only (%) | Total  Nº offspring (mean) |
| --- | --- | --- | --- | --- | --- | --- |
| Mothers |  |  |  |  |  |  |
| Proportion boys |  |  |  |  |  |  |
| < 0.5 | 232,459 | 1941 | 31,773 | 1968 | 68 | 2.6 |
| >= 0.5 | 428,554 | 1942 | 56,847 | 1968 | 66 | 2.5 |
| *Total* | *661,013* | *1942* | *88,620* | *1968* | *67* | *2,5* |
|  |  |  |  |  |  |  |
| First offspring |  |  |  |  |  |  |
| Girl | 321,915 | 1941 | 42,636 | 1968 | 67 | 2.5 |
| Boy | 339,927 | 1941 | 44,980 | 1968 | 67 | 2.5 |
|  |  |  |  |  |  |  |
| First two offspring |  |  |  |  |  |  |
| Girl-girl | 132,471 | 1941 | 16,764 | 1968 | 67 | 2.8 |
| Boy-girl or girl-boy | 282,158 | 1941 | 35,978 | 1968 | 67 | 2.7 |
| Boy-boy | 148,048 | 1941 | 19,122 | 1968 | 67 | 2.8 |
|  |  |  |  |  |  |  |
| Fathers |  |  |  |  |  |  |
| Proportion boys |  |  |  |  |  |  |
| < 0.5 | 243,996 | 1939 | 67,357 | 1969 | 56 | 2.6 |
| >= 0.5 | 447,128 | 1939 | 119,059 | 1969 | 55 | 2.5 |
| *Total* | *691,124* | *1939* | *186,416* | *1969* | *56* | *2.5* |
|  |  |  |  |  |  |  |
| First offspring |  |  |  |  |  |  |
| Girl | 335,533 | 1939 | 90,300 | 1969 | 56 | 2.5 |
| Boy | 355,591 | 1939 | 96,116 | 1969 | 56 | 2.5 |
|  |  |  |  |  |  |  |
| First two offspring |  |  |  |  |  |  |
| Girl-girl | 137,578 | 1939 | 36,477 | 1969 | 55 | 2.8 |
| Boy-girl or girl-boy | 293,147 | 1939 | 77,090 | 1969 | 55 | 2.7 |
| Boy-boy | 153,790 | 1939 | 40,724 | 1969 | 55 | 2.8 |

SUPPLEMENTARY TABLE 2. Age adjusted hazard ratio of death among mothers (n=661,013) born 1925-54 according to

Nº boys in offspring with follow up of death from age 50 among those having at least two or more offspring.

|  | Hazard ratio (95 % CI) | | | | | |
| --- | --- | --- | --- | --- | --- | --- |
|  | All causes (n=93,518) | | Cardiovascular (n=22,072) | | Lung cancer and COPD  (n= 11,503) | |
|  | Model 1^a^ | Model 2^b^ | Model 1^a^ | Model 2^b^ | Model 1^a^ | Model 2^b^ |
| Nº boys |  |  |  |  |  |  |
| 0 (reference) | 1.00 | 1.00 | 1.00 | 1.00 | 1.00 | 1.00 |
| 1 | 0.96 (0.94-0.98) | 0.92 (0.91-0.94) | 0.97 (0.93-1.01) | 0.95 (0.91-0.99) | 0.96 (0.91-1.01) | 0.91 (0.87-0.96) |
| 2 | 0.91 (0.89-0.93) | 0.85 (0.84-0.87) | 0.94 (0.90-0.98) | 0.90 (0.86-0.94) | 0.88 (0.83-0.93) | 0.81 (0.76-0.86) |
| 3 | 0.91 (0.88-0.93) | 0.84 (0.82-0.87) | 0.97 (0.91-1.02) | 0.91 (0.86-0.96) | 0.91 (0.85-0.99) | 0.82 (0.76-0.89) |
| 4 | 0.94 (0.86-0.97) | 0.86 (0.82-0.91) | 1.12(1.02-1.22) | 1.03 (0.94-1.12) | 0.96 (0.84-1.09) | 0.84 (0.74-0.96) |
| 5 | 1.04 (0.95-1.14) | 0.95 (0.87-1.04) | 1.24(1.06-1.46) | 1.13 (0.96-1.32) | 0.99 (0.77-1.28) | 0.86 (0.67-1.11) |
| 6 or more | 1.11 (0.96-1.29) | 1.05 (0.91-1.22) | 1.52(1.20-1.94) | 1.40 (1.10-1.79) | 0.90 (0.57-1.42) | 0.80 (0.51-1.26) |
| *Per boy* | *0.98 (0.97-0.99)* | *0.95 (0.95-0-96)* | *1.01(0.99-1.02)* | *1.00 (0.99-1.02)* | *0.97 (0.95-0.99)* | *0.94 (0.92-0.96)* |
| *Quad term (p-value)* ^c^ | *<0.001* | *<0.001* | *<0.001* | *<0.001* | *0.006* | *0.001* |
| *Mothers vs fathers (p-value)*^d^ | *< 0.001* | *< 0.001* | *0.512* | *0.139* | *< 0.001* | *0.001* |
|  |  |  |  |  |  |  |
| Nº girls |  |  |  |  |  |  |
| 0 (reference) | 1.00 | 1.00 | 1.00 | 1.00 | 1.00 | 1.00 |
| 1 | 0.94 (0.92-0.95) | 0.91 (0.89-0.93) | 0.91 (0.88-0.95) | 0.90 (0.87-0.94) | 0.91 (0.87-0.96) | 0.87 (0.83-0.92) |
| 2 | 0.89 (0.87-0.90) | 0.84 (0.82-0.86) | 0.87 (0.83-0.90) | 0.85 (0.81-0.88) | 0.88 (0.84-0.93) | 0.82 (0.77-0.87) |
| 3 | 0.87 (0.85-0.90) | 0.82 (0.80-0.84) | 0.89 (0.84-0.94) | 0.86 (0.81-0.91) | 0.88 (0.81-0.95) | 0.79 (0.73-0.86) |
| 4 | 0.91 (0.86-0.95) | 0.84 (0.80-0.88) | 1.00 (0.91-1.09) | 0.93 (0.85-1.03) | 0.90 (0.78-1.04) | 0.80 (0.69-0.91) |
| 5 | 0.92 (0.83-1.02) | 0.86 (0.78-0.96) | 1.08 (0.90-1.30) | 1.01 (0.84-1.21) | 0.76 (0.56-1.04) | 0.68 (0.49-0.92) |
| 6 or more | 0.89 (0.74-1.07) | 0.85 (0.71-1.01) | 1.29 (0.97-1.71) | 1.19 (0.89-1.57) | 0.90 (0.55-1.48) | 0.81 (0.49-1.32) |
| *Per girl* | 0.96 (0.95-0.97) | 0.94 (0.94-0.95) | 0.98 (0.96-0.99) | 0.96 (0.95-0.98) | 0.96 (0.94-0.98) | 0.93 (0.91-0.95) |
| *Quad term (p-value)* ^c^ | *<0.001* | *<0.001* | *<0.001* | *<0.001* | *0.024* | *0.004* |
| *Girls vs boys (p-value)* ^d^ | *0.0052* | *0.0042* | *0.0028* | *0.0022* | *0.5728* | *0.5398* |
| *Mothers vs fathers (p-value)*^d^ | *< 0.001* | *< 0.001* | *0.002* | *0.029* | *< 0.001* | *< 0.001* |

^a^ Model 1: Adjusting for age

^b^ Model 2: Adjusting for age, number of opposite sex and length of education

^c^ Test of quadratic term for number of boys or girls respectively

^d^ Test of difference in effect for the linear term for number of boys and number of girls

SUPPLEMENTARY TABLE 3. Age adjusted hazard ratio of death among fathers (n= 691,124) born 1925-54 according to Nº boys in offspring

with follow up of death from age 50 among those having at least two or more offspring.

|  | Hazard ratio (95 % CI) | | | | | |
| --- | --- | --- | --- | --- | --- | --- |
|  | All causes (n= 196,867) | | Cardiovascular (n=75,557) | | Lung cancer and COPD  (n= 21,994) | |
|  | Model 1^a^ | Model 2^b^ | Model 1^a^ | Model 2^b^ | Model 1^a^ | Model 2^b^ |
| Nº boys |  |  |  |  |  |  |
| 0 (reference) | 1.00 | 1.00 | 1.00 | 1.00 | 1.00 | 1.00 |
| 1 | 0.96 (0.95-0.98) | 0.96 (0.95-0.97) | 0.98 (0.96-1.00) | 0.98 (0.96-1.00) | 0.97 (0.94-1.01) | 0.98 (0.94-1.02) |
| 2 | 0.96 (0.95-0.97) | 0.95 (0.94-0.97) | 0.98 (0.95-1.00) | 0.98 (0.96-1.00) | 0.97 (0.93-1.01) | 0.97 (0.93-1.02) |
| 3 | 1.00 (0.98-1.02) | 0.98 (0.96-1.00) | 1.04 (1.01-1.07) | 1.03 (1.00-1.06) | 1.04 (0.99-1.10) | 1.02 (0.97-1.08) |
| 4 | 1.03 (1.00-1.07) | 0.99 (0.96-1.03) | 1.10 (1.05-1.16) | 1.06 (1.01-1.12) | 1.11 (1.01-1.21) | 1.05 (0.96-1.14) |
| 5 | 1.06 (1.00-1.13) | 1.01 (0.95-1.07) | 1.11 (1.01-1.22) | 1.05 (0.96-1.15) | 0.84 (0.69-1.02) | 0.77 (0.63-0.94) |
| 6 or more | 1.07 (0.97-1.18) | 1.01 (0.91-1.11) | 1.18 (1.02-1.37) | 1.10 (0.95-1.27) | 1.09 (0.81-1.46) | 0.98 (0.74-1.31) |
| *Per boy* | 1.00 (1.00-1.01) | 1.00 (1.00-1.01) | 1.02 (1.01-1.02) | 1.01 (1.00-1.02) | 1.01 (1.00-1.02) | 1.00 (0.99-1.01) |
| *Quad term (p-value)* ^c^ | *<0.001* | *<0.001* | *<0.001* | *<0.001* | *0.065* | *0*.625 |
|  |  |  |  |  |  |  |
| Nº girls |  |  |  |  |  |  |
| 0 (reference) | 1.00 | 1.00 | 1.00 | 1.00 | 1.00 | 1.00 |
| 1 | 0.97 (0.96-0.98) | 0.97 (0.96-0.98) | 0.97 (0.95-0.99) | 0.98 (0.96-1.00) | 0.98 (0.94-1.01) | 0.98 (0.94-1.02) |
| 2 | 0.97 (0.96-0.98) | 0.96 (0.95-0.98) | 0.98 (0.96-1.00) | 0.99 (0.96-1.01) | 0.99 (0.95-1.03) | 0.99 (0.95-1.03) |
| 3 | 0.98 (0.96-1.00) | 0.96 (0.95-0.98) | 1.01 (0.98-1.04) | 1.00 (0.97-1.03) | 1.02 (0.97-1.08) | 1.00 (0.95-1.06) |
| 4 | 1.05 (1.01-1.08) | 1.01 (0.98-1.04) | 1.11 (1.06-1.17) | 1.08 (1.02-1.13) | 1.10 (1.00-1.20) | 1.04 (0.95-1.14) |
| 5 | 1.03 (0.97-1.11) | 0.98 (0.92-1.05) | 1.03 (0.93-1.14) | 0.97 (0.88-1.08) | 1.18 (0.98-1.41) | 1.08 (0.90-1.29) |
| 6 or more | 1.16 (1.04-1.28) | 1.09 (0.98-1.22) | 1.30 (1.12-1.52) | 1.22 (1.04-1.42) | 0.83 (0.57-1.21) | 0.76 (0.52-1.10) |
| *Per girl* | 1.00(1.00-1.01) | 0.99 (0.99-1.00) | 1.01 (1.00-1.02) | 1.01 (1.00-1.01) | 1.01 (1.00-1.03) | 1.00 (0.99-1.02) |
| *Quad term (p-value)* ^c^ | *<0.001* | *<0.001* | *<0.001* | *<0.001* | *0.035* | *0.398* |
| *Girls vs boys (p-value)* ^d^ | *0.5024* | *0.4606* | *0.3959* | *0.3596* | *0.8354* | *0.8519* |

^a^ Model 1: Adjusting for age

^b^ Model 2: Adjusting for age, number of opposite sex offspring and length of education

^c^ Test of quadratic term for number of boys or girls respectively

^d^ Test of difference in effect for number of boys and number of girls

SUPPLEMENTARY TABLE 4. Age adjusted means and proportions of some risk factors among mothers according to number of boys and girls among those having more than one offspring in a linked sub cohort (n=50,736) being part of the CONOR

|  | Age at examin-ation  (sd) | BMI  (kg/m ^2^) | Tri- glycerides  (mmol/l) | Chol-  esterol  (mmol/l) | Systolic blood pressure  (mmHg) | Diabetes  (%) | Mental distress  (%) | Daily smoker  (%) | Alcohol  (>2units per week) | Physical  Inactivity  (%) | Family history of CHD  (%) | Cardiovascular disase  (%) | N |
| --- | --- | --- | --- | --- | --- | --- | --- | --- | --- | --- | --- | --- | --- |
| Nº boys |  |  |  |  |  |  |  |  |  |  |  |  |  |
| 0 | 51 (10) | 25.7 | 1.49 | 5.93 | 131.6 | 2.3 | 5.5 | 36.3 | 10.0 | 6.0 | 49.8 | 4.1 | 10,183 |
| 1 | 52 (10) | 25.8 | 1.51 | 5.95 | 132.1 | 2.2 | 5.5 | 34.7 | 9.8 | 5.9 | 49.8 | 4.2 | 21,628 |
| 2 | 52 (10) | 26.0 | 1.52 | 6.01 | 133.0 | 2.3 | 5.2 | 32.9 | 8.2 | 6.3 | 50.3 | 4.8 | 13,629 |
| 3 | 54 (11) | 26.7 | 1.63 | 6.13 | 135.4 | 3.5 | 5.6 | 31.5 | 5.4 | 8.2 | 50.7 | 6.6 | 4157 |
| 4 | 57 (11) | 27.5 | 1.70 | 6.31 | 139.0 | 5.0 | 5.3 | 31.2 | 2.7 | 11.7 | 54.2 | 7.0 | 909 |
| 5 | 59 (11) | 28.4 | 1.88 | 6.46 | 130.0 | 8.1 | 4.5 | 27.3 | 0.6 | 13.5 | 56.1 | 13.7 | 178 |
| 6 or more | 61 (10) | 30.1 | 2.02 | 6.29 | 148.6 | 10.2 | 5.7 | 26.9 | 0 | 18.4 | 44.4 | 14.0 | 52 |
| *P-value* | *<0.001* | *<0.001* | *<0.001* | *<0.001* | *0.001* | *<0.001* | *<0.001* | *<0.001* | *<0.001* | *<0.001* | *<0.001* | *0.188* |  |
|  |  |  |  |  |  |  |  |  |  |  |  |  |  |
| Nº girls |  |  |  |  |  |  |  |  |  |  |  |  |  |
| 0 | 51 (10) | 25.8 | 1.48 | 5.94 | 131.7 | 2.2 | 5.6 | 35.6 | 9.9 | 6.1 | 49.6 | 4.4 | 11,270 |
| 1 | 51 (10) | 25.8 | 1.50 | 5.97 | 132.3 | 2.2 | 5.5 | 34.8 | 9.5 | 5.9 | 50.4 | 4.2 | 22,114 |
| 2 | 54 (10) | 26.0 | 1.54 | 5.99 | 132.8 | 2.4 | 5.1 | 32.3 | 8.2 | 6.4 | 49.2 | 4.4 | 12,576 |
| 3 | 54 (11) | 26.8 | 1.64 | 6.08 | 135.8 | 3.7 | 5.4 | 33.0 | 6.0 | 8.1 | 51.3 | 6.9 | 3708 |
| 4 | 57 (10) | 27.3 | 1.77 | 6.35 | 138.0 | 4.0 | 6.6 | 29.4 | 2.1 | 12.0 | 56.4 | 8.9 | 863 |
| 5 | 59 (10) | 27.6 | 1.85 | 6.38 | 142.6 | 10.8 | 3.7 | 24.1 | 2.1 | 13.2 | 43.1 | 10.8 | 160 |
| 6 or more | 63 (10) | 28.5 | 2.02 | 6.57 | 140.0 | 4.6 | 12.1 | 31.8 | 2.3 | 14.3 | 57.9 | 4.6 | 45 |
| *P-value* | *<0.001* | *<0.001* | *<0.001* | *<0.001* | *0.001* | *0.486* | *<0.001* | *<0.001* | *<0.001* | *<0.001* | *<0.001* | *0.176* |  |
| *Missing* |  |  |  |  |  |  |  |  |  |  |  |  |  |

SUPPLEMENTARY TABLE 5. Age adjusted means and proportions of some risk factors among fathers according to number of boys and girls among those having more than one offspring in a linked sub cohort (n=44,794) being part of the CONOR

|  | Age at examin-ation  (sd) | BMI  (kg/m ^2^) | Tri- glycerides  (mmol/l) | Chol-  esterol  (mmol/l) | Systolic blood pressure  (mmHg) | Diabetes  (%) | Mental distress  (%) | Daily smoker  (%) | Alcohol  (>2units per week) | Family history of CHD  (%) | Cardiovascular disase  (%) | Physical  Inactivity  (%) | N |
| --- | --- | --- | --- | --- | --- | --- | --- | --- | --- | --- | --- | --- | --- |
| Nº boys |  |  |  |  |  |  |  |  |  |  |  |  |  |
| 0 | 55 (12) | 26.6 | 2.00 | 6.00 | 139.0 | 3.2 | 3.9 | 31.3 | 20.4 | 45.9 | 11.0 | 6.5 | 9349 |
| 1 | 56 (12) | 26.7 | 1.97 | 5.99 | 139.8 | 3.6 | 3.5 | 31.3 | 20.5 | 45.9 | 12.2 | 6.4 | 19,528 |
| 2 | 56 (11) | 26.6 | 1.98 | 6.00 | 139.8 | 3.8 | 3.3 | 29.6 | 19.1 | 46.0 | 12.9 | 6.3 | 11,591 |
| 3 | 58 (11) | 26.8 | 2.00 | 6.01 | 140.9 | 4.7 | 3.5 | 31.4 | 15.4 | 47.7 | 15.1 | 6.9 | 3424 |
| 4 | 59 (10) | 27.1 | 2.01 | 6.05 | 142.0 | 4.9 | 4.8 | 30.2 | 12.6 | 49.1 | 21.2 | 10.3 | 718 |
| 5 | 61 (10) | 27.2 | 2.17 | 6.04 | 144.0 | 6.8 | 4.0 | 31.1 | 6.5 | 51.2 | 16.3 | 12.8 | 148 |
| 6 or more | 64 (10) | 27.2 | 2.55 | 6.25 | 144.9 | 0 | 9.1 | 27.8 | 6.1 | 40.6 | 27.8 | 20.0 | 36 |
| *P-value* | *<0.001* | *<0.001* | *0.428* | *0.084* | *<0.001* | *0.242* | *<0.001* | *0.081* | *<0.001* | *0.003* | *<0.001* | *<0.001* |  |
|  |  |  |  |  |  |  |  |  |  |  |  |  |  |
| Nº girls |  |  |  |  |  |  |  |  |  |  |  |  |  |
| 0 | 55 (11) | 26.6 | 1.97 | 5.99 | 139.0 | 3.5 | 3.9 | 31.2 | 20.6 | 46.0 | 11.5 | 5.9 | 10,278 |
| 1 | 56 (11) | 26.6 | 1.98 | 5.99 | 139.1 | 3.7 | 3.6 | 31.3 | 20.3 | 46.1 | 12.2 | 6.4 | 19,759 |
| 2 | 56 (11) | 26.6 | 2.00 | 6.01 | 139.6 | 3.6 | 3.2 | 29.4 | 18.9 | 46.6 | 13.0 | 6.7 | 10,906 |
| 3 | 58 (11) | 26.7 | 1.98 | 6.05 | 140.3 | 4.0 | 3.2 | 31.2 | 15.9 | 45.7 | 14.2 | 7.9 | 3040 |
| 4 | 60 (11) | 27.2 | 2.01 | 6.09 | 142.4 | 6.1 | 4.2 | 31.4 | 11.6 | 47.9 | 20.6 | 12.2 | 654 |
| 5 | 62 (10) | 27.0 | 1.98 | 6.20 | 143.2 | 7.6 | 7.1 | 32.1 | 7.7 | 39.7 | 26.3 | 9.9 | 134 |
| 6 or more | 63 (10) | 26.9 | 1.62 | 6.23 | 142.8 | 4.4 | 5.3 | 39.1 | 0 | 63.2 | 17.4 | 14.3 | 23 |
| *P-value* | *<0.001* | *0.001* | *0.111* | *0.002* | *<0.001* | *0.019* | *0.003* | *0.257* | *<0.001* | *0.097* | *0.001* | *<0.001* |  |

SUPPLEMENTARY TABLE 6. Age adjusted hazard ratio of all cause and CVD mortality in sub-strata of the main cohort

|  | Interpregnancy interval highest quartile | | Having had twins  ^a^ | | Experienced perinatal death ^b^ | | Having had pre-eclampsia ^b^ | |
| --- | --- | --- | --- | --- | --- | --- | --- | --- |
|  | All cause | CVD | All cause | CVD | All cause | CVD | All cause | CVD |
| Mothers |  |  |  |  |  |  |  |  |
| *Sex of first offspring* |  |  |  |  |  |  |  |  |
| Girl | ref | ref | ref | ref | ref | ref | ref | ref |
| Boy | 1.00 (0.97-1.03) | 1.04 (0.99-1.09) | 1.07 (0.97-1.19) | 1.20 (0.99-1.47) | 1.08 (0.99-1.16) | 1.10 (0.91-1.33) | 0.90 (0.80-1.02) | 0.96 (0.76-1.22) |
| *P-value (different effect*  *from the others in sample)* | *0.094* | *0.986* | *0.001* | *0.112* | *0.106* | *0.530* | *0.071* | *0.511* |
|  |  |  |  |  |  |  |  |  |
| *Proportion boys* |  |  |  |  |  |  |  |  |
| Trend effect | 1.03 (1.01-1.06) | 1.06 (1.01-1.11) | 1.13 (0.98-1.31) | 1.51 (1.13-2.03) | 1.19 (1.05-1.35) | 1.34 (0.99-1.83) | 0.81 (0.67-0.98) | 0.81 (0.56-1.18) |
| *P-value (different effect*  *from the others in sample)* | *0.611* | *0.419* | *0.217* | *0.026* | *0.005* | *0.124* | *0.028* | *0.162* |
| Fathers |  |  |  |  |  |  |  |  |
| *Sex of first offspring* |  |  |  |  |  |  |  |  |
| Girl | ref | ref | ref | ref | ref | ref | ref | ref |
| Boy | 1.01 (0.99-1.02) | 1.00 (0.97-1.03) | 0.98 (0.92-1.04) | 1.06 (0.97-1.17) | 0.98 (0.93-1.04) | 1.01 (0.92-1.10) | 1.01 (0.93-1.10) | 1.01 (0.84-1.16) |
| *P-value (different effect*  *from the others in sample)* | *0.114* | *0.761* | *0.493* | *0.255* | *0.621* | *0.812* | *0.222* | *0.813* |
|  |  |  |  |  |  |  |  |  |
| *Proportion boys* |  |  |  |  |  |  |  |  |
| Trend effect | 1.02 (1.99-1.05) | 1.01 (0.97-1.07) | 1.01 (0.93-1.10) | 1.10 (0.97-1.25) | 1.06 (0.97-1.15) | 1.11 (0.96-1.28) | 1.04 (0.92-1.18) | 0.97 (0.79-1.19) |
| *P-value (different effect*  *from the others in sample)* | *0.020* | *<0.001* | *0.005* | *0.007* | *0.003* | *0.064* | *0.482* | *0.230* |

^a^ Twins were identified as two births in the same month and year (zygosity unknown) (n=18,995 mothers) and (n=15,952 fathers)

^b^ These were among those who become parents after the Medical Birth Registry was established in 1967 (n=548,930 mothers) and (n=552,143 fathers)
